# Relationship between Personality Profiles and Suicide Attempt via Medicine Poisoning among Hospitalized Patients: A Case-Control Study

**DOI:** 10.1155/2014/675480

**Published:** 2014-11-20

**Authors:** Ali Reza Shafiee-Kandjani, Shahrokh Amiri, Asghar Arfaie, Azadeh Ahmadi, Mahmoud Farvareshi

**Affiliations:** ^1^Clinical Psychiatry Research Center, Department of Psychiatry, Tabriz University of Medical Sciences, Tabriz, Iran; ^2^Child and Adolescent Psychiatry, Clinical Psychiatry Research Center, Department of Psychiatry, Tabriz University of Medical Sciences, Tabriz, Iran; ^3^Department of Psychiatry, Razi Mental Hospital, El Goli Boulevard, P.O. Box 5456, Tabriz 51677, Iran; ^4^Department of Psychiatry, Tabriz University of Medical Sciences, Tabriz, Iran; ^5^Tabriz University of Medical Sciences, Tabriz, Iran

## Abstract

*Objectives*. Inflexible personality traits play an important role in the development of maladaptive behaviors among patients who attempt suicide. This study was conducted to investigate the relationship between personality profiles and suicide attempt via medicine poisoning among the patients hospitalized in a public hospital. *Materials and Methods*. Fifty-nine patients who attempted suicide for the first time and hospitalized in the poisoning ward were selected as the experimental group. Sixty-three patients hospitalized in the other wards for a variety of reasons were selected as the adjusted control group. Millon Clinical Multiaxial Personality Inventory, 3rd version (MCMI-III) was used to assess the personality profiles. *Results*. The majority of the suicide attempters were low-level graduates (67.8% versus 47.1%, OR = 2.36). 79.7% of the suicide attempters were suffering from at least one maladaptive personality profile. The most common maladaptive personality profiles among the suicide attempters were depressive personality disorder (40.7%) and histrionic personality disorder (32.2%). Among the syndromes the most common ones were anxiety clinical syndrome (23.7%) and major depression (23.7%). *Conclusion*. Major depression clinical syndrome, histrionic personality disorder, anxiety clinical syndrome, and depressive personality disorder are among the predicators of first suicide attempts for the patients hospitalized in the public hospital due to the medicine poisoning.

## 1. Introduction

Given that suicide is considered as an essential psychological and social problem, there is a universal attempt to prevent it. The prevalence of contemplating suicide is 16% [1] and suicide attempt is 4.4% [2] during one's life. The risk of death from suicide is 30–40 times more for the suicide attempters than normal population [3, 4]. Furthermore, the likelihood of death among patients with repetitive self-harm behaviors is 100 times more than general population. One suicide attempt per second and one death per 40 seconds due to suicide have been reported [5]. The rate of suicide in Iran is lower than Western societies; however, in comparison with Middle East countries it is considered high. Recently suicide attempt has gone up to 9.4 per 100,000 in Iran. Furthermore, the age of people who die from suicide has been declining (under 40) [6]. Prevention of suicide or suicidal attempt in especial psychiatric patients with or without previous attempt(s) needs indicative and selective interventions. More risk factors lead to increased likelihood of mortality and morbidity among these cases [7].

Suicide is considered as a medical problem for the public healthcare services [8]; mortality and morbidity may be increased leading to more treatment and rehabilitation costs when the medicine poisoning is the main cause of the suicidal attempts in a community. Almost 55.8% of suicide attempts in Iran occur by medicine poisoning [6]. Suicide attempt is among the highest predicators for committing suicide [3, 9]. Thus, the diagnosis of the intervening predicators in suicide attempts where medicine poisoning is involved might have an applied significance. The studies show that committing suicide is a multifactor practice and there is no unique factor to prevent it [5]. A variety of biological, social, and personal predisposing factors are introduced as the risk factors for suicide [10]. Various psychiatric disorders have been proposed as the intervening factors in suicide attempts [3, 11]. Given the fact that personality affects our emotional and behavioral patterns, it is assumed that personality profile can be employed to prevent the risk of attempts at suicide [12, 13]. Temperament traits may play an important role in the prediction of potential suicidal risk especially in patients with mood disorders as explained by Pompili et al. [14].

Studies on the records of psychiatric patients with and without suicide attempt show that those who committed suicide possessed anger, aggression, anxiety, and depression personality profiles [12]. Based on a study, depressed patients with borderline personality traits were characteristically vulnerable and had familial generalized anxiety disorder in comparison with other groups [15]. The study on suicide attempters in Portuguese public hospital refers to depression, suicidal ideation and intention, onset of major depression, namely, hopelessness, pessimism, interpersonal relations, and life events as the influential factors in suicide. A review-study on patients with borderline personality disorder reports the likelihood of suicide attempt as 5–10%, four hundred times greater than public population. Researchers estimate that 40–85% out of borderline patients have several suicide attempts and self-mutilation is one of the risk factors among these cases [16]. A study in Switzerland on opium users and alcohol consumers shows nonfatal overdose records. Furthermore, there is relation among suicide attempts, violence, and nonfatal overdose records [17]. The results of a study indicate that 21% of suicide attempters were alcohol dependent. The rate of alcohol consumption, psychological/medical problems, and emotional/sexual misuses is high among suicide attempters [18].

The increase of suicide rate and the use of medicine poisoning method by the suicide attempters in Iran [6] as well as the scarcity of studies on the relationship between personality profile and suicide attempt using medicine poisoning were the main reasons for the present study [18]. Considering personality profiles may provide us with precise aspects of suicide attempts. The current study aims to explore the relationship between personality profiles and committing suicide via medicine poisoning method among the hospitalized patients in the University hospital of Sina in Tabriz, Iran, using the Millon Clinical Multiaxial Inventory third version (MCM-III).

## 2. Materials and Methods

The study is a case-control design. Subjects were selected through the convenient sampling method from among the patients hospitalized in the poisoning ward of Sina University hospital, Tabriz, Iran. The data collection procedure was carried out from the 1 May to 1 September, 2013.

### 2.1. Participants

Altogether 127 patients participated in the study. Fifty-nine (39 male and 25 female) suicide attempters with no previous suicide records were selected as the experimental group. Sixty-eight normal patients (38 male and 30 female) who were hospitalized in the surgical and internal wards with no suicide records were put in the control group. The two groups were matched for gender and age variables. The estimated age mean was 28.28 ± 6.83. The youngest patient was 18 years old and the oldest one was 50 years old.

### 2.2. Inclusion and Exclusion Criteria

The inclusion criteria are

- participants aged 18 and elder,
- patients admitted because of medicine poisoning suicide attempt,
- first attempt,
- at least 7th grade education level,
- patients' written informed consent.

The exclusion criteria included the following conditions:

- a history of previous suicide attempt record,
- a history of using psychiatric medicines,
- having severe physical/mental disabilities,
- suffering from another medical condition such as epilepsy and cardiovascular diseases.

Those patients who had not completed the questionnaire in full were eliminated from the study. A written informed consent was introduced addressing all ethical issues requested by the university ethical committee. In this study the patients unwilling to participate were excluded. In addition, every patient could leave the study whenever they would like to do so.

### 2.3. Instruments

Millon Clinical Multiaxial Inventory, third version (MCMI-III), is composed of 175 Yes/No items. It is intended for adults (18/18+). It assesses the interaction of Axis I and Axis II disorders based on the DMS-IV classification system. It is modeled on 4 scales: (1) eleven personality clinical scales: schizoid, avoidant, depressive, dependent, histrionic, narcissistic, antisocial, sadistic, compulsive, negativistic, and masochistic, (2) three severe personality pathology scales: schizotypal, borderline, and paranoid, (3) seven clinical syndrome scales: anxiety, somatoform, bipolar, dysthymia, alcohol dependence, drug dependence, and posttraumatic stress disorder, and (4) three severe clinical syndrome scales: thought disorder, major depression, and delusional disorder.

Correction scales are used to detect careless, confused, and random responding. The 3 modifying indices used for correction scales are disclosure, desirability, and debasement. Item scoring is compatible with the symptoms of clinical indices with the range of 1–3. The Farsi version of MCMI-III is validated using Cronbach's alpha method. The reported validity was 0.79–0.94. The positive predictive powers ranged from 0.92 to 0.98 and the negative predictive powers ranged from 0.93 to 0.99; also overall predictive powers ranged from 0.58 to 0.83 for all scales [19].

### 2.4. Procedure

In pursuing the study such ethical considerations as participants' consent, confidentiality of the participants' private and personal information, and no interference in the control and experimental groups' affairs were observed. All participants were tested in their second-fifth day of hospitalization. MCMI-III questionnaires were filled in via a face-to-face structured interview. It was scored and interpreted later by a psychologist. Demographic information was collected via a questionnaire, including the respondents' personal information as age, gender, educational level, career, marital status, educational degree, suicide history, date of suicide, history of other medical conditions, or psychiatric medication record. The demographic questionnaire was completed through interview with a patient or his/her family members and the patient's medical file.

### 2.5. Data Analysis

The data was analyzed using SPSS version 17. The independent *t*-test was used to do the comparison among personality profile means. In order to predict the likelihood of the membership in each group, discrimination analysis applying stepwise was employed. The Chi-square test and Fisher's exact test were run to find out the relationship between the variables and groups where the *P* value < 0.01 is considered significant.

## 3. Results

One hundred and twenty-seven participants took part in the study, 59 in the experimental group and 68 in the control group. The gender distribution frequency in the experimental group was 34 for men (57.6%) and 25 (42.4%) for women. It was 38 (55.9%) for male and 30 (44.1%) for female in the control group. Fisher's exact test shows no significant difference between experimental and control groups regarding gender distribution frequency (*X*2 = 0.03, df = 1, *P* = 0.085). The participants' estimated age mean in the experimental group was *M* = 27.86, SD = 6.77 and in the control group was *M* = 28.63, SD = 6.92. The results of the independent *t*-test showed no significant difference between age mean in two groups (*t* = 0.63, df = 125, *P* = 0.53). The age range for the 35 (65.5%) suicide attempters was 18–30. According to Fisher's exact test, 34 (57.6%) patients were recorded as single and 25 (42.4%) patients were married in the experimental group, whereas in the control group 35 (51.5%) patients were single and 33 (48.5%) patients were married ones. However, no significant difference in gender frequency distribution was revealed. Regarding the educational level of the participants, in the experimental group 40 (67.8%) participants had diploma/under diploma degrees and 19 (32.2%) participants were university graduates. In the control group, 32 (47.1%) participants had diploma/secondary school degrees and 36 (52.9%) participants were university graduates. The results of the Chi-square test show that the educational level of the majority of suicide attempters was under diploma (*x*2 = 5.32, df = 1, *P* = 0.02); OR (95% CI) = 2.36 (1.14–4.88). Regarding the employment conditions of the participants, in the experimental group 7 (10.3%) participants were jobless, 15 (22.1%) participants were businessmen, 19 (27.9%) participants were university students, 17 (25%) participants were housewives, and 10 (14.7%) participants were clerks. In the control group, 12 (20.3%) participants were jobless, 9 (15.3%) participants were businessmen, 13 (22%) participants were university students, 16 (27.1%) participants were housewives, and 9 (15.3%) participants were clerks/employees. The results of the Chi-square test showed no significant difference between two groups' job distribution frequency (*x*2 = 3.40, df = 4, *P* = 0.49). In order to determine the distribution frequency of personality profiles among participants, the scores given to each item are added up and the cutting score was used to diagnose the patients with personality disorder. As illustrated in Table 1 the investigation of the personality disorder prevalence in MCMI-III scale between the experimental and control groups showed that among suicide attempters 12 (20.3%) patients with no personality disorder were recorded. Whereas 13 (22%) patients show just one facet of maladaptive personality, 34 (57.6%) patients had two/more facets of personality disorder. However, in the control group (patients with no suicide attempt record) 54 (79.4%) patients with no personality disorder were recorded, while 9 (13.2%) patients presented just one facet of maladaptive personality disorder and 5 (7.4%) patients presented two/more facets of the personality disorder. According to the results of the Chi-square test, the distribution frequency of the personality disorder among suicide attempters was higher than among patients with no suicide attempt record. In total, 79.7% of the suicide attempters suffered from at least one personality disorder. While the highest personality problems among suicide attempters were depression (40.7%), histrionic (32.2%), anxiety (23.7%), and major depression (23.7%), the lowest are alcohol dependency and masochistic.

Table 1 presents the prevalence of other personality disorders. In order to compare the unadjusted personality profiles between two groups, we added up the scores of each item in MCMI-III subscales. Then the independent *t*-test was used to measure the mean of the experimental and control groups, the results of which show a difference between the two groups regarding the mean of syndrome 22 indices of maladaptive personality profiles (*P* < 0.001). In other words, suicide medicine overdose patients suffered from more symptoms at

- three severe clinical syndrome scales,
- seven clinical syndrome scales,
- three severe personality pathology scales,
- nine personality clinical scales.

There was no significant difference between the mean of syndrome scores for two personality profiles of narcissistic and compulsive.

To discriminate the experimental and control groups on the basis of the mean scores of MCMI-III scales, the discrimination analysis of stepwise type was used. The results of this statistical interpretation method to discriminate between the patients with and without suicide attempt record showed that in the first step the clinical disorder of major depression type, in the second step the major depression and histrionic, and in the third step the major depression, histrionic, anxiety, and depressive clinical personality are the discriminating factors between two groups. Using four maladaptive personality profiles for the participants, (i.e., depressive, anxiety, histrionic, and major depression), we discriminated 43 (79.9%) patients out of 59 as suicide attempters in the experimental group. Furthermore, in the control group 61 (89.7%) patients out of 68 with no suicide attempt record were discriminated using the four maladaptive personality profiles. In total, approximately 81.9% of the established personality profiles can be used to discriminate among the patients with and without suicide attempt records via overdose drug usage.

## 4. Discussion

The results of this experimental study which aimed to find out the relationship between personality profiles and the first attempt at suicide via medicine poisoning among the patients hospitalized in a University hospital in Tabriz, Iran, show that the age range among suicide attempters was 18–30 which supports the report of the study conducted in Iran [6]. However, it is not in accordance with the results of the studies conducted in the Western societies where the emphasis is on the higher age range of suicide attempters [18, 20]. Bearing in mind that Iran is among the youngest societies in the world and Iranian youth face various social problems, it is not surprising that Iranian suicide attempters are mainly young people.

The findings of our study show that 79.7% of suicide attempters have at least one maladaptive personality disorder. In a similar vein, as the study performed by Cavanagh and colleagues showed that more than 90% of patients who died because of suicide suffered from psychological disorder [21], the inclusion and exclusion criteria used in the present study and the selection of participants from among those patients who attempted suicide by taking overdose drug for the first time account for the percentage incompatibility between this study and the previous ones. The patients with other problems who were not considered in the study might be another explanation for the aforementioned discrepancy [22].

According to the results, the suicide attempters' mean scores were higher than nonattempters in all scales except for compulsive and narcissistic ones. It is in agreement with the study in which the majority of suicide attempters had maladaptive personality profiles [23]. Neuroticism is the first and the most influential factor in the personality profile [13].

The majority of the suicide attempters in this study were single which supports the findings declared by Kposowa [24]. In other words, there is a relationship between suicide and marital status of the patients. However, the results of the studies [18, 20] show that the majority of suicide attempters were married which accounts for the social conditions where these studies were carried out. In addition, due to the fact that suicide is often committed in the early adulthood [6], the probability of suicide attempt for the married ones is low in Iran.

This study did not reveal any relationship between the employment and suicide attempt. However, some studies reported such relations [25–27]. This is because of the fact that the majority of the participants in this study were young and single and most of them were either students or housewives. Thus, further complementary researches are needed with more population to obtain precise and accurate results about the relationship between employment and suicide attempt in Iran.

In line with the previous study the educational levels of the suicide attempters were high school and diploma degree [20]. The majority of the suicide attempters were high school graduates or with higher educations. It could be interpreted that individuals with low educational levels are emotion-oriented rather than mind-oriented as they face life problems. They lose the chances to solve their problems via problem-solving strategies that are more efficient.

As the previous studies report, the suicide attempters were suffering from family problems, social isolation, and interpersonal problems [28]. Although the mean of syndrome scores for the patients with an maladaptive personality was higher than suicide attempters via overdose medicine use, just the four criteria, that is, severe major depression clinical syndrome, histrionic personality disorder, anxiety clinical syndrome, and depressive clinical personality, were the discriminators of the patients who were hospitalized for medicine poisoning and those who committed suicide via overdose medicine use. In line with the previous studies [12, 15, 29, 30] severe major depression clinical syndrome and depressive clinical personality are among the risk factors for suicide attempt. A systematic review conducted in Iran indicates that the rate of depression among suicide attempters ranges from 36% to 83% [6]. A research on the depressed individuals shows that 1 out of 4 had nonfatal suicide attempt during lifetime [31]. The results of a study show that depression increases the risk of contemplating suicide but it is not considered as a risk factor for committing suicide [23]. The prevalence of contemplating suicide among depressed patients is high. As it is reported in a study 25% of depressed patients experienced suicide contemplating in the past two weeks [32]. The contradictions on the relation between depression and suicide can be attributed to the features of the sample population selected for the study. As it was observed in the present study, the suicide attempters were suffering from depression disorder as well as depressive personality disorder syndromes. Laget and the colleagues showed that the rate of suicide attempts among depressed patients as their personality profile was high [33]. Thus, an efficient medical intervention is needed to help the suicide attempter suffering from depression symptoms.

According to the present study histrionic personality disorder is a predicator for suicide attempt which is contrary to the study conducted by Craig and Bivens [34]. The previous reports show that the prevalence of histrionic personality disorder among suicide attempters was 1.8% [20]. It seems that committing suicide is a call by suicide attempters for attention getting.

In line with the previous studies, anxiety clinical syndrome has been among the risk factors for suicide attempt via medicine poisoning in the hospitalized patients [12, 15, 23, 35]. Among the individuals with a lifetime history of suicide attempt, 29.5% were suffering from anxiety disorder [20]. In other words, the comorbid personality disorder and anxiety disorder increase the contribution of anxiety as a risk factor for suicide attempt. It is assumed that the stressors cause increased anxiety and depression which in the long run directs one's attention towards committing suicide [36].

In accordance with a study, no relationship between suicide attempt and alcohol/drug dependency was revealed [37]. However, some studies show such a relation [20, 30, 35, 38]. Another study emphasizes on the alcohol/drug consumption as a risk factor for suicide attempt among teenagers [1]. Given the fact that drug resistance is high among the drug consumers, they may have overdose use [39].

Unlike some studies in which borderline personality disorder was not among the predicators of suicide attempt [16, 40, 41], various inclusion criteria may lead to the contradiction among the findings. According to this study the social and geographical conditions may affect the age features of the suicide attempters which in turn may cause different pathological conditions. Thus, in reporting the findings of any study the social conditions as well as the inclusion and exclusion criteria should be taken into account. Furthermore, the future studies should pay more attention to the seriousness of the maladaptive personality profiles which contributes to the diagnosis of the various aspects of psychological pathology of the suicide attempters.

## 5. Conclusions

The results show that the majority of the suicide attempters were single, young, and diploma/under diploma graduates. On the basis of MCMI-III 79.7% suicide attempters were suffering from at least one personality disorder. Depression was the first manifestation of maladaptive personality disorder among suicide attempters in Iran. The least common personality disorder pertains to alcohol-dependent and masochistic patients. Four maladaptive personality disorders including clinical syndrome of major depression, histrionic personality disorder, anxiety clinical syndrome, and depressive clinical personality were among the predicators of suicide attempts which should be taken into consideration in sampling suicide attempters in the future studies. There is a need for further complementary studies to emphasize on the effects of maladaptive personality profiles on suicide. Owing to the personality vulnerability of the suicide attempters, it is necessary to deal with their mood and anxiety problems. Thus, simultaneous psychotherapy and medication are necessary to treat maladaptive personality. In addition, there is a need for complementary studies to plan specific programs to manage the suicide attempters' treatment.

## 6. Limitations

The limitations of the current study were low sample size, selection bias through studying at one hospital, and cross-sectional design which may influence the application of the results. These limitations should be considered in the future studies.

## Figures and Tables

**Table 1:** *t*-test result of personality profile comparing SDOP and control groups and the prevalence of personality problems.

MCMI-III scales	control	SDOP	*t*-value	control	SDOP
	Mean (SD)	Mean (SD)		*N* (%)	*N* (%)
Personality clinical scales					
Schizoid	7.19 (4.25)	11.12 (4.79)	4.89^**^	1 (1.5)	1 (1.7)
Avoidant	4.96 (3.59)	10.32 (5.75)	6.38^**^	0	9 (15.3)
Depressive	6.37 (5.11)	12.78 (6.13)	6.42^**^	5 (7.4)	24 (40.7)
Dependent	7.87 (3.47)	12.14 (4.75)	5.82^**^	1 (1.5)	13 (22)
Histrionic	9.41 (4.13)	11.90 (4.08)	3.39^*^	5 (7.4)	19 (32.2)
Narcissistic	13.44 (3.36)	12.78 (5.04)	0.88	2 (2.9)	4 (6.8)
Antisocial	6.22 (3.83)	9.07 (3.79)	4.19^**^	0	1 (1.7)
Sadistic	7.21 (4.28)	11.29 (5.08)	4.91^**^	0	1 (1.7)
Compulsive	14.82 (3.32)	14.73 (3.83)	0.15	5 (7.4)	4 (6.8)
Negativistic	8.04 (4.90)	14.05 (5.50)	6.50^**^	2 (2.9)	7 (11.9)
Masochistic	5.38 (3.18)	9.25 (4.14)	5.94^**^	0	0
Severe personality pathology scales					
Schizotypal	4.07 (3.98)	9.10 (6.18)	5.51^**^	0	2 (3.4)
Borderline	5.50 (3.48)	11.14 (5.08)	7.36^**^	0	2 (3.4)
Paranoid	7.04 (4.21)	11.68 (5.15)	5.56^**^	0	2 (3.4)
Clinical syndrome scales					
Anxiety	3.19 (2.92)	8.83 (5.56)	7.27^**^	0	14 (23.7)
Somatoform	2.74 (3.13)	7.93 (4.72)	7.39^**^	0	2 (3.4)
Bipolar	3.43 (2.91)	6.86 (3.62)	5.92^**^	0	1 (1.7)
Dysthymia	4.31 (4.36)	10.78 (5.85)	7.11^**^	3 (4.4)	10 (16.9)
Alcohol dependence	3.40 (2.00)	5.02 (2.46)	4.09^**^	0	0
Drug dependence	3.49 (2.38)	4.71 (2.63)	2.75^*^	0	1 (1.7)
Posttraumatic stress disorder	3.15 (3.40)	8.93 (5.69)	7.04^**^	1 (1.5)	2 (3.4)
Severe clinical syndrome scales					
Thought disorder	4.82 (3.76)	10.15 (5.87)	6.16^**^	1 (1.5)	11 (18.6)
Major depression	4 (4.44)	11.73 (6.61)	7.81^**^	0	14 (23.7)
Delusional disorder	3.04 (2.70)	6.14 (4.17)	5.01^**^	0	3 (5.1)

df = 125, ^*^ *P* < 0.01, ^**^ *P* < 0.001, suicidal drug overdose patients (SDOP).
